# Ramifications of Heat Stress on Rabbit Production and Role of Nutraceuticals in Alleviating Its Negative Impacts: An Updated Review

**DOI:** 10.3390/antiox12071407

**Published:** 2023-07-11

**Authors:** Tarek A. Ebeid, Hamad S. Aljabeili, Ibrahim H. Al-Homidan, Zdeněk Volek, Hassan Barakat

**Affiliations:** 1 Department of Animal Production and Breeding, College of Agriculture and Veterinary Medicine, Qassim University, Buraydah 51452, Saudi Arabia; jbiely@qu.edu.sa (H.S.A.); homiedan@qu.edu.sa (I.H.A.-H.); 2 Department of Poultry Production, Faculty of Agriculture, Kafrelsheikh University, Kafr El-Sheikh 33516, Egypt; 3 Department of Physiology of Nutrition and Product Quality, Institute of Animal Science, Přátelství 815, 10400 Prague, Czech Republic; volek.zdenek@vuzv.cz; 4 Department of Microbiology, Nutrition and Dietetics, Faculty of Agrobiology, Food and Natural Resources, Czech University of Life Sciences Prague, 16500 Prague, Czech Republic; 5 Department of Food Science and Human Nutrition, College of Agriculture and Veterinary Medicine, Qassim University, Buraydah 51452, Saudi Arabia; 6 Food Technology Department, Faculty of Agriculture, Benha University, Moshtohor 13736, Egypt

**Keywords:** *Oryctolagus cuniculus*, high ambient temperature, nutrition, performance, intestinal histomorphology, immunity

## Abstract

Heat stress has become a widespread concern worldwide, which is a major environmental stress that causes substantial economic loss in the rabbit industry. Compared to other agricultural animals, rabbits are more sensitive to heat stress as they have fewer sweat glands and a thicker coat of fur, increasing the heat dissipation complexity. Thus, heat stress hurts rabbits’ productivity, meat quality, reproductive performance, antioxidative properties, immune responsiveness, intestinal histomorphology, and microbiome. Nutraceuticals include vitamins, minerals, antioxidants, organic acids, fatty acids, probiotics, prebiotics, synbiotics, enzymes, and medicinal plants due to the possible impacts on maintaining common biological situations, strengthening immune response, and preventing illness, which ultimately led to an increase in productivity. Nutraceuticals have recently attracted a lot of attention to alleviate the adverse impacts of heat stress in rabbit farms. The objective of the current review is to provide acquaintance with the recent findings about the impact of heat stress on rabbit productivity and the advantages of dietary supplementation of nutraceuticals in mitigating it.

## 1. Introduction

**Figure 1:**
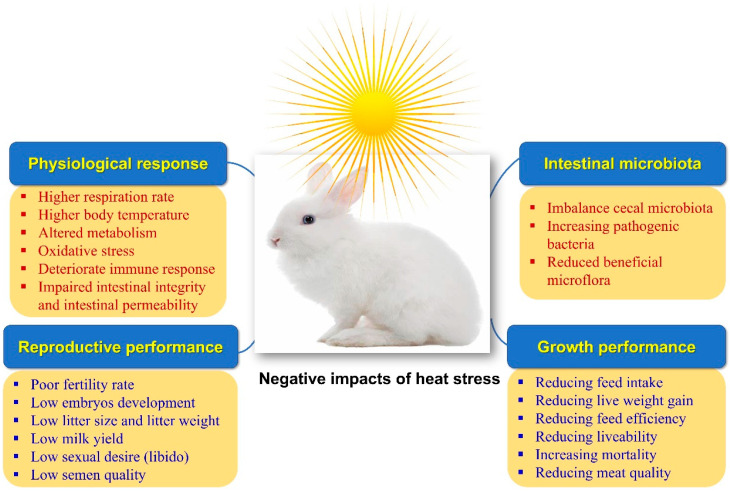
Impacts of heat stress on rabbits’ physiological response, productive and growth performance, and intestinal microbiota.

Rabbits are raised primarily for their meat, hair, and fur. Rabbit meat is characterized by low contents of fat, cholesterol, and sodium. At the same time, it is rich in protein, polyunsaturated fatty acids (PUFA), minerals (potassium, phosphorus, and selenium), and vitamins (B_12_ and niacin) [1]. With the worsening of climate change and the global warming phenomena, heat stress (HS) has become one of the most important types of stress that are challenging the rabbit industry, especially in hot and semi-hot regions of the world. The whole world is now suffering from high ambient temperatures, which have recently reached levels not seen before. HS is the highest serious stress and threat to the rabbit and poultry industry [2,3]. Rabbits are more susceptible to HS than other agricultural animals as they own fewer sweat glands and a thicker coat of fur, increasing the complexity of heat scattering [4,5]. Moreover, the genetically improved rabbits are characterized by rapid growth and higher metabolic rates, increasing their susceptibility to HS [6]. Thus, HS leads to significant economic losses in rabbit production as it elevates body temperature and disturbs normal physiological status, deteriorating growth performance, meat characteristics, reproductive traits, antioxidative properties, and immune responsiveness [7,8] (Figure 1).

**Figure 2:**
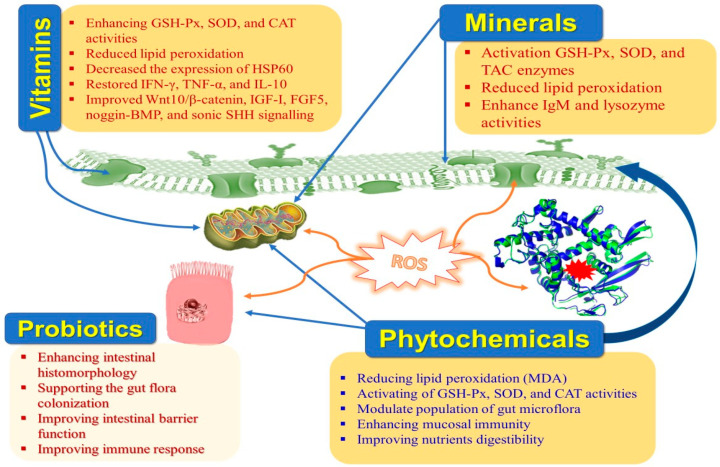
Main impacts of nutraceuticals on heat-stressed rabbits.

Moreover, HS hurts the intestinal histomorphology and microbiome in rabbits [9]. The negative effects of HS on rabbit productivity can be mitigated with the help of cooling systems, ventilation, and management strategies. Implementing cutting-edge technology into the building infrastructure can be challenging under extreme conditions. Thus, nutritional manipulation to relieve the unfavorable influences of HS is an effective additional approach [10]. Nutraceuticals are dietary components that offer additional health benefits that override their nutritional benefits. Due to their potential impacts on maintaining normal physiological situations, strengthening the immune system, and preventing illness—which ultimately lead to an increase in productivity—nutraceuticals have recently attracted a lot of attention in rabbit farms (Table 1 and Figure 2). Nutraceuticals include vitamins, minerals, antioxidants, organic acids, fatty acids, probiotics, prebiotics, synbiotics, enzymes, medicinal plants, etc. [11]. Natural antioxidants are crucial in safeguarding the animal against the damage caused by free radicals. Weaned rabbits become extremely vulnerable to enteric infections due to the prevention of using antibiotics as growth enhancers because they have a very complicated and distinctive digestive system [12,13].

Moreover, there is a growing interest in natural alternatives to antibiotics that could be used in rabbit production and antibiotic-free rabbit meat. Several dietary supplements—including vitamins, minerals, and enzymes—are already utilized to preserve the normal physiological status, support immunological responsiveness, and improve rabbit productivity in thermo-neutral and HS circumstances [14,15,16,17]. It has been suggested that probiotics, prebiotics, synbiotics, and organic acids could replace antibiotic growth enhancers in rabbit production because they promote a healthy intestinal environment [18,19,20,21]. Furthermore, phytobiotics or phytogenics are being utilized more frequently in rabbit nutrition as antioxidants, physiological stimulants, flavorings, digestive aids, and colorants, and for protecting and treating different pathological troubles [22,23,24]. The objective of the current review is to give information on the recent findings about the influence of HS on rabbit productivity and the advantages of dietary supplementation of nutraceuticals in alleviating it.

## 2. Effect of HS on Growth Performance

It is well known that HS is the highest hazard factor deteriorating growing rabbits’ growth performance and viability [4,8,25,26,27,28,29]. Farghly et al. [8] reported that rabbits were susceptible to HS, which resulted in deteriorating growth performance indicators in terms of body weight (BW), body weight gain (BWG), feed intake (FI), and feed conversion ratio (FCR). Similarly, Matics et al. [3] noted that HS negatively influenced the growing rabbits’ FI, BWG, BW, and fat deposits. During HS circumstances, rabbits attempt to disperse the extra heat generated inside the body by reducing FI, and this FI decline could be about 28–38% [4,25]. Besides, under HS conditions, growing rabbits prefer to direct energy toward heat dissipation rather than the growth and building of muscles and tissues [30]. Moreover, HS suppresses the hypothalamus’s appetite–satiety center and enhances leptin and adiponectin secretion, decreasing FI [30,31].

Furthermore, elevating the ambient temperature reduced digestion [32] and absorption of nutrients [33], which, coupled with decreasing FI, almost resulted in reducing the supply of essential nutrients, leading to a deteriorating growth rate, final BW, meat quality traits, antioxidative status, and immune responsiveness in fattening rabbits [4,34]. Sirotkin et al. [26] pointed out that exposure to HS resulted in suppressing growth parameters (FI, FCR, and viability), reducing serum insulin-like growth factor 1 (IGF-I) content, and increasing serum corticosterone concentration and mortality of growing rabbits. From another point of view, several studies elucidated that HS harmed thyroid activity in the form of reducing serum concentrations of triiodothyronine (T_3_) and thyroxine (T_4_), which resulted in retardation of protein synthesis and an increase of protein destruction, leading to the suppression of the growth rate in growing rabbits [29,35].

## 3. Effect of HS on Reproductive Performance

In general, HS negatively impacted reproductive performance in female and male rabbits, which posed a danger to the rabbit business in hot and semi-hot climates [36,37]. Rabbits subjected to HS conditions had a decrease in fertility, embryo development, litter size, litter weight, and milk production [28,37]. Marco-Jimenez et al. [37] reported that maternal exposure to high environmental temperatures decreased litter weight, litter size, and kit weight at birth, while the stillborn rate was greater in heat-stressed does during pregnancy.

**Table 1:** Nutraceuticals’ potential to alleviate heat stress impacts in rabbit production.

Additives	Level	Heat Stress Conditions	Animal	Main Impacts	References
Vitamin C	0.5 g/kg dietfrom 5 to 14 wk of age	32.44 °C and 84.67% relative humidity	Giant Flander male growing rabbits	Enhanced feed intake and feed conversion ratio. Improved serum concentration of SOD and TAC. Reduced serum concentrations of triglycerides, hydrogen peroxide, and MDA. Decreased Staphylococcus aureus, E. coli, and total bacterial counts in cecum.	[38]
Vitamin C	200 mg/kg diet from 6 to 12 wk of age	28–39 °C and 60% relative humidity	New Zealand White growing rabbits	Improved body weight gain and feed conversion ratio. Restored cortisol, leptin, IFN-γ, TNF-α, and IL-10 plasma concentrations to normal levels. Decreased HSP70 and forkhead box P3 (FOXP3) gene expression in liver and kidney tissues.	[39]
Vitamin E	0.25 g/kg diet from 7 to 14 wk of age	36.4 °C and 97% relative humidity	Californian unsexed growing rabbits	Improved body weight, weight gain, feed intake, and feed conversion ratio. Improved carcass yield % and total edible parts %.	[40]
Vitamin E	100 mg/kg diet for 3 months	32.9 °C and 80.38% relative humidity	New Zealand White rabbits does	Improved pregnancy rate and litter size. Improved serum GSH, GSH-Px, SOD, CAT, GST, and TAC. Enhanced lysozyme, IgG, and IgM concentrations.	[41]
Vitamin A	12,000 IU/kg diet	30–34 °C	Rex rabbits	Increased hair follicle density and fur quality. Regulated Wnt10/β-catenin, insulin-like growth factor-1 (IGF-I), fibroblast growth factor 5 (FGF5), noggin-BMP, and sonic hedgehog (SHH).	[42]
Selenium	25 and 50 mg of nano-Se/kg diet from 7 to 13 wk of age	33 °C and 90% relative humidity	Domestic rowing rabbits	Enhanced live body weight, body weight gain, feed intake, and feed conversion ratio. Enhanced carcass %. Improved serum GSH and SOD concentrations. Regulated inflammatory cytokines (IFNγ and IL-4).	[43]
Selenium	0.3 mg organic Se/kg diet for 12 wk	31 °C and 75% relative humidity	Adult V-line male rabbits	Decreased rectal temperature and respiration rate. Decreased MDA concentration in seminal plasma. Enhanced TAC concentration in seminal plasma. Enhanced total functional sperm counts and percentages of integrated sperm membranes.	[44]
Zinc	20, 40, 60, and 80 mg nano-Zn/kg dietfor 60 d	38.20–40.10 °C and 45–50% relative humidity	New Zealand White male growing rabbits	Enhanced body weight, weight gain, feed intake, and conversion ratio. Improved carcass% and hind parts %. Reduced carcass fat %. Reduced meat lipid oxidation (TBARS content). Improved water-holding capacity.	[16]
Zinc	75 mg ZnSO_4_/kg diet or 75 mg Zn picolinate/kg diet from 32 to 42 wk of age	30.7–37.6 °C and 70–80% relative humidity	New Zealand White rabbit bucks	Decreased serum concentration of AST, ALT, and glucose. Improved antioxidative status (serum concentration of SOD and TAC). The increased serum concentration of testosterone.	[45]
Copper	200 mg Cu-methionine/kg diet or 200 mg copper-glycine/kg diet for 5 wk	30.12 °C and 82.40% relative humidity	V line unsexed growing rabbits	Enhanced body weight, body weight gain, and feed conversion ratio. Improved serum concentrations of TAC, GSH-Px, and SOD. Decreased serum MDA concentration. Increased serum concentrations of HDL-cholesterol.	[46]
Copper	100 mg Cu-acetate/kg diet or 50 mg nano-Cu/kg diet from 5 to 14 wk of age	17 and 22 °C	New Zealand White unsexed growing rabbits	Improved final body weight, body weight gain, and feed conversion ratio. Enhanced nutrient digestibility (nitrogen-free extract, dry, and organic matter). Enhanced serum concentrations of complement component-3, IgM, and lysozyme activity.	[47]
Chromium	0.4–1.6 mg organic Cr/kg diet	30 °C and 80% relative humidity	Growing rabbits	Improved final body weight, body weight gain, and feed intake. Enhanced cell-mediated immunity.	[48]
Chromium	2.5 mg Cr-yeast/kg diet for 198 d	29.3 °C and 71% relative humidity	New Zealand White male rabbits	Improved final body weight, body weight gain, and feed conversion ratio. Enhanced serum testosterone level. Improved advanced-sperm motility, live spermatozoa, and morphologically normal spermatozoa.	[49]
Probiotics	5 × 10^6^ CFU *Clostridium butyricum*, 2 × 10^8^ CFU *Enterococcus faecium*, or 2.5 × 10^6^ CFU *C. butyricum* + 1 × 10^8^ CFU *E. faecium*/kg diet from 35 to 91 d of age	31.78 °C and 60.19% relative humidity	New Zealand White male growing rabbits	Enhanced final body weight, body weight gain, and feed conversion ratio. Improved serum concentrations of total protein, globulin, HDL-cholesterol, complement component 3, and lysozyme activity. Improved cecal fermentation (cecal concentrations of total volatile fatty acids and butyric acids). Improved duodenal histomorphometry indices (higher villus height, thicker muscular layer thickness, lower crypt depth, and lower villus width).	[50]
Probiotics	3 × 10^9^ CFU *Saccharomyces cerevisiae*/kg or 3 × 10^9^ CFU *Lactobacillus acidophilus*/kg from 5 to 13 wk of age	33 °C and 81% relative humidity	New Zealand White growing rabbits	Enhanced final body weight, body weight gain, and feed conversion ratio. Supported the beneficial yeast species (*Yarrowia lipolytica*). Reduced pathogenic bacteria (*Salmonella* spp., *C.* spp., and *Enterobacteria* spp.).	[51]
Prebiotics	0.3% mannan-oligosaccharides or 0.05% isomalto-oligosaccharide from 6 to 16 wk of age	19 °C	New Zealand White male growing rabbits	Improved final body weight and feed intake. Enhanced carcass criteria (dressing % and giblets %). Increased duodenal villi area and length. Increased spleen white bulb area and length. Elevated liver GSH-Px, insulin-like growth factor 1, SOD gene expression, and muscle insulin-like growth factor 1 receptor expression. Reduced spleen IL-6 expression gene.	[19]
Prebiotics	3 g Bio-Mos^®^/kg diet from 4 to 12 wk of age	31.50 °C and 79.07% relative humidity	New Zealand White male growing rabbits	Improved final body weight, body weight gain, and feed conversion ratio. Increased serum levels of hemoglobin, total protein, globulin, and albumin. Decreased respiration rate, rectal temperature, and heart rate.	[7]
*Moringa oleifera*	200 mg *Moringa oleifera* leaves powder/kg BW daily for 4 wk (from 32 to 32 wk of age)	35 °C and 80% relative humidity	New Zealand White male rabbits	Decreased rectal temperature and respiration rate. Enhanced intestinal histomorphology. Down-regulated the mRNA expression of TNF-α, HSP-A2, GSH-Px, and IL-1α. Elevated IL-6 expression. Decreased jejunal contents of lipopolysaccharide, pro-inflammatory cytokines, and myeloperoxidase. Elevated mRNA expression of tight junction proteins. Reduced MDA concentration in jejunal mucosa. Modulated intestinal microbiota composition positively. Enhanced mucosal immunity (down-regulation of gene expression of TNFRSF13C, LBP, and COX2, while up-regulation of the gene expression of protein digestion and absorption pathway, including PRSS2, LOC100349163, CPA1, CPB1, SLC9A3, SLC1A1, and SLC7A9).	[52,53]
*Moringa oleifera*	50 mg *Moringa oleifera* leaves ethanolic extract/kg BW for 12 consecutive weeks	31.11 °C and 87% relative humidity	V-line rabbit bucks	Reduced rectal temperature. Increased serum total antioxidant capacity concentration. Increased sperm concentration, sperm progressive motility, sperm viability, sperm normal morphology, intact acrosome sperm, and sperm with the integrated cell membrane.	[54]
Ginger	7.5 g ginger powder/kg diet from 5 to 13 wk of age	33 °C and 74.5% relative humidity	APRI growing rabbits	Enhanced final body weight and feed conversion ratio. Increased carcass weight. Enhanced digestibility of dry matter, organic matter, crude protein, and nitrogen-free extract. Minimized serum levels of triglycerides, total cholesterol, HDL-cholesterol, and LDL-cholesterol. Increased plasma concentration of total antioxidant capacity. Reduced plasma concentration of MDA.	[55]
Ginger	250 mg ginger/doe/d for 8 wk	35 °C and 80% relative humidity	New Zealand White virgin female rabbits	Decreased rectal temperature, skin temperature, ear temperature, and respiration rate. Improved body weight and feed intake. Enhanced the reproductive traits (conception rate, litter size, and litter weight at birthing and weaning). Increased plasma concentrations of progesterone, T3, T4, total protein, albumin, and globulin. Decreased plasma concentrations of cortisol, urea, and creatinine.	[56]
Thyme	16 g thyme/kg diet for 90 d	39 °C and 30–35% relative humidity	New Zealand White male rabbits	Enhanced body weight gain, feed intake, and feed conversion ratio. Increased the ejaculate volume, sperm viability, and sperm motility. Reduced serum concentrations of serum ALT, AST, urea, and creatinine.	[57]
Thyme	100 thyme essential oil mg/kg diet from 6 to 9 months of age	33 °C and 80% relative humidity	APRI rabbit does	Improved reproductive performance (milk production, ovulation rate, total litter size, live litter size at birth, litter size at weaning, viability rate at birth, and viability rate at weaning). Enhanced antioxidative status (increased TAC and GSH). Decreased serum lipid peroxidation (MDA concentration). Improved serum concentrations of immunoglobulins (IgG and IgM).	[58]
Turmeric	2.5 g turmeric nanoparticles/kg diet from 5 to 13 wk of age	32.77 °C and 43.23% relative humidity	APRI growing rabbits	Improved final body weight, body weight gain, and feed conversion ratio. Reduced serum concentrations of total lipids, total cholesterol, triglycerides, and LDL-cholesterol, while increasing HDL-cholesterol. Increased serum immunoglobulins. Improved antioxidative status (serum concentrations of TAC, SOD, GSH-Px, and GSH). Reduced liver lipid peroxidation (MDA concentration).	[59]
Maca (*Lepidium meyenii*)	400 or 600 mg maca extract/head twice weekly	33 °C and 80% relative humidity	V-line rabbit bucks	Enhanced live body weight, feed intake, and feed conversion ratio Reduced serum concentration of total bilirubin, creatinine, glucose, cholesterol, and triglycerides. Enhanced cortisol and testosterone levels. Improved antioxidative status (serum concentration of TAC and SOD). Decreased serum lipid peroxidation (MDA concentration). Improved semen quality parameters.	[24]
Pumpkin	2 mL pumpkin seed essential oil/kg diet from 5 to 13 wk of age	38 °C and 60% relative humidity	New Zealand White male growing rabbits	Improved body weight, weight gain, feed intake, and conversion ratio. Increased serum levels of total protein, albumin, and globulin. Lowered plasma total bilirubin, ALT, AST, creatinine, uric acid, glucose, cortisol, T3, and corticosterone concentrations. Reduced anti-inflammation agent (exhibiting negative caspase-3 immunoreactivity surrounding portal tract).	[60]

SOD-Superoxide dismutase, TAC-total antioxidant capacity, IFN-γ-interferon-gamma, TNF-α- tumor necrosis factor-alpha, IL-10-interleukin 10, HSP70-heat shock protein 70, GSH-glutathione, GSH-Px-glutathione peroxidase, CAT- catalase, GST-glutathione S-transferase, MDA- Malondialdehyde, TBARS-thiobarbituric acid reactive substances, ALT- Alanine aminotransferase, AST- Aspartate transaminase, T3-triiodothyronine, T4-thyroxine. García and Argente [61] elucidated that rabbits’ dams exposed to HS had minor ovulation rate, normal embryo %, embryo quality, zona pellucida thickness, and later embryogenesis. Several studies documented that a long exposure to thermal stress resulted in oxidative stress, consequently deteriorating rabbit females’ endocrine system and ovarian physiological functions [26,62]. It was noted that exposure to HS reduced the relative weight of the ovary [62,63]. Additionally, exposure to HS resulted in the impairment of ovarian granulosa cells’ responsiveness, reducing plasma progesterone concentration, destructing the ovarian cell nucleoli, and weakening the responsiveness of ovarian granulosa cells to follicle-stimulating hormone (FSH) in rabbit does [26]. Moreover, HS increased mortality in both adult mothers and offspring and deteriorated offspring growth [26]. Tang et al. [63] pointed out that HS had a negative influence on the physiological performance of female rabbits in terms of ovary weight % and plasma contents of interleukin (IL)-2, IL-8, catalase (CAT), and glutathione peroxidase (GSH-Px), and accelerated ovarian apoptosis and unhealthy follicles, as well as altered miRNAs expression in rabbit does. The CAT and GSH-Px play a vital role as antioxidative enzymes in rabbits’ earlier phases of folliculogenesis; therefore, exposure to HS suppressed ovarian folliculogenesis, maturation, and ovulation [64].

Regarding gene expression, Marco-Jiménez et al. [65] explained that the harmful effect of HS on live-born kits and litter size is attributed to a disruption in gene expression pattern, including the up-regulation of vascular endothelial growth factor (VEGF) and octamer-binding transcription factor 4 (OCT-4), and the down-regulation of Ifn-γ in endometrial tissue and embryos leading to retardation of fetal development during gestation. From another point of view, HS during pregnancy and lactation might affect the fetal growth and postnatal development of newborn kits through the lactation period [26,37,66]. This retardation in postnatal development might be attributed to HS hurting milk yield [25,31]. Therefore, kits suckled less milk, reducing weight gain [25].

Male rabbits are more oversensitive to HS than other male farm animals [67]. HS deteriorated sexual desire (libido) and semen quality characteristics, including concentration, motility, viability, morphological parameters, and metabolic activity [68,69]. Maya-Soriano et al. [67] reported that rabbit bucks subjected to HS (30 °C) had a lower sperm total motility, progressive motility, and specific motility parameters. Sabés-Alsina et al. [68] documented that HS had a negative effect on rabbit spermatozoa parameters, including viability %, acrosome abnormalities %, presence of distal cytoplasmic droplets %, motility parameters (total motility %, curvilinear velocity, average path velocity), and sperm metabolic activity. The exposure of male rabbits to HS reduced viable spermatozoa % and increased acrosome abnormalities % [69]. HS affects male rabbits with temporary infertility or sub-fertility and libido during the hot season [70,71]. Under high ambient temperatures, hypothalamic GnRH secretion is blocked, significantly impacting spermatogenesis and testicular function, and lowering semen quality in male rabbits [72]. HS also causes oxidative stress and reactive oxygen species (ROS) generation that damages sperm’s DNA, mitochondrial, and smooth endoplasmic reticulum, altering its cytoskeleton and axoneme and decreasing motility [73,74]. In testis, the excellent result of HS is a devastation of spermatogonia and the later stages of spermatogenesis by apoptosis and histomorphological modifications in seminiferous tubules and seminiferous epithelium in male rabbits [71].

## 4. Effect of HS on Carcass Traits and Meat Quality

Carcass characteristics and meat quality parameters of rabbits are very important criteria for consumer acceptance. Several studies elucidated that HS negatively affected carcass and meat quality traits [3,66]. Matics et al. [4] noted that a high ambient temperature hurt slaughter weight, hot carcass weight, chilled carcass weight, and reference carcass weight in growing rabbits. Zeferino et al. [66] observed that HS reduced slaughter weight, carcass weight, and relative weights of internal organs (thoracic viscera, liver, and kidneys), decreasing meat juiciness and meat color (redness and yellowness) while increasing cooking loss. HS did not influence other meat quality characteristics, including pH (24 h and 48 h), water-holding capacity, and the Warner–Bratzler force [66]. Contrarily, Dahmani et al. [27] postulated that HS did not significantly influence the carcass yield %, forelegs %, hind legs %, and loin %. In contrast, the liver %, kidney %, peritoneal fat %, and inter-scapular fat % were reduced in fattening rabbits. Similarly, Matics et al. [4] noted that HS harmed perirenal and scapular fat percentages in growing rabbits. Additionally, Zeferino et al. [66] concluded that heat-stressed rabbits had lower fat depots. Meanwhile, Liu et al. [29] elucidated that chronic HS decreased the liver index (%), while the shoulder fat % and kidney fat % were increased.

## 5. Effect of HS on the Intestinal Microbiome

The intestinal microbiome performs a vital task in gut action and health and is involved in nutrient digestion, immune response, and productiveness [75,76]. The highest percentage of digestive disorders is noticed in juvenile rabbits, mostly during the weaning period (feed transmission, handling, stressful factors, etc.). Such disturbances might be linked to unbalance and instability in intestinal microbiota and the inability of nonspecific and specific immune responses to combat harmful pathogens effectively [77]. Environmental stressors, mainly HS, can modify the balance of the gut flora in growing rabbits [78,79,80]. Bai et al. [79] postulated that thermal stress increased the number of Firmicutes, Proteobacteria, and Verrucomicrobiota at the phylum class, while reducing the *Bacteriodota* number in growing rabbits.

Liu et al. [29] reported that HS affected the cecal microflora and increased the number of cecal *Proteobacteria* of *Proteus*, and reduced the number of *Lachnospiraceae*, *Ruminococcaceae*, and *Candidatus saccharimonas*, which may lead to inflammatory diseases in growing rabbits. Yasoob et al. [81] demonstrated that HS induced an imbalance in cecal microbiota by increasing the quantity of *Proteobacteria* in growing rabbits. El-Badawi et al. [9] noted that HS increased the total count of pathogenic bacteria such as *Salmonella*, *E. coli*, *Staphylococcus aureus*, *Clostridium perfringens*, and molds in the small intestine and cecum of growing rabbits. Moreover, Patra and Kar [82] documented that HS causes injury to the mucosal epithelia’s structure and deteriorates the intestinal barrier function, increasing intestinal permeability to toxins and pathogens in farm animals. This damage increased the sensitivity to oxidative stress insults and inflammation.

## 6. Effect of HS on Antioxidative Properties

Under thermoneutral conditions, there is an equilibrium between the generation and elimination of free radicals (e.g., ROS) by the antioxidative system. Enzymes such as superoxide dismutase (SOD), glutathione peroxidase (GSH-Px), and catalase (CAT) scavenge reactive oxygen species (ROS), which are neutralized by the antioxidative defence system [83]. While under HS conditions, the redox balance is disturbed, and consequently, the ROS generation is elevated, leading to oxidative stress in rabbits [62,79,84]. Saghir et al. [34] indicated that exposure to HS resulted in reducing the activities of antioxidative enzymes, including GSH-Px, SOD, and CAT, and increasing serum oxidative markers such as protein carbonyl (as an index of amino acids oxidation) and malondialdehyde (MDA, as an index of lipids peroxidation) in growing rabbits. Similarly, several studies revealed that the plasma concentration of GSH-Px, SOD, and CAT was significantly minimized, while the plasma level of MDA was elevated in heat-stressed rabbits [84,85]. Likewise, Madkour et al. [86] observed that HS at 36 °C lessened the amounts of SOD, GSH, and CAT, and elevated the MDA concentration in the blood plasma and muscle of broiler rabbits. Bai et al. [79] postulated that the plasma total antioxidant capacity (TAC) concentration was reduced in growing rabbits under HS conditions. Moreover, thermal stress hurts metabolism by increasing the contents of 4-pyridoxic acid, kynurenine, 20-OH-leukotriene B_4_, and dopamine. It reduces pyridoxal’s value, making rabbits susceptible to inflammatory and oxidative stress [79]. Yasoob et al. [81] noted that HS generated cecal oxidative stress, whereas the MDA concentration in cecal mucosa was elevated in growing rabbits subjected to HS conditions. Madkour et al. [87] elucidated that HS had an unfavorable effect on hepatic antioxidative status, including reduced glutathione (GSH), SOD, and CAT concentrations, while the MDA concentration was increased in fattening rabbits. In female rabbits, Mutwedu et al. [62] demonstrated that exposing rabbits to HS (35–36 °C) caused oxidative stress, reduced the renal values of CAT, SOD, and GSH-Px, and raised the renal content of MDA.

## 7. Effect of HS on Immune Responsiveness

Inhibition of humoral and cell-mediated immune responses was seen in rabbits exposed to cyclic or chronic HS [87,88]. HS inhibits the immune system components and disturbed homeostasis in rabbits [89]. Liu et al. [29] noted that HS harmed growing rabbits’ thymus index (%). Saghir et al. [34] indicated that HS induced the raising of pro-inflammatory cytokines containing tumor necrosis factor-α (TNF-α), IL-1β, and interferon-gamma (IFNγ) in growing rabbits. These results conform with reports postulated that HS stimulated inflammatory signaling, including TNF-α, IL-1β, and IFNγ in heat-stressed rabbits [43,79,87,89]. Yasoob et al. [81] noted that HS adversely affected the mucosal immune response and increased cecal concentrations of TNF-α, IL-1α, and IL-1β as markers of cecal mucosa inflammation in growing rabbits. Additionally, in fattening rabbits exposed to HS, the serum lysosome activity and nitric oxide levels were reduced [34]. Moreover, HS disturbed the equilibrium between anti-inflammatory and pro-inflammatory cytokines [34], which might be connected with a progressive inflammation response [86]. Abdel-Latif et al. [39] observed that HS had a negative influence on IFN-γ, TNF-α, and heat shock protein 70 (HSP70) expression leading to affect the infiltration of regulatory T cells adversely and NK cells in New Zealand White (NZW) growing rabbits. From another point of view, normal thyroid hormone concentrations are essential for the proper function of the immune system [35]. Exposure to HS suppresses the hypothalamic–pituitary–thyroid axis and reduces the serum concentrations of T_3_ and T_4_ [29,61], and, finally, depressing the immune response in growing rabbits. Furthermore, considering the link between oxidative stress and inflammation, it might be indicated that rabbits exposed to HS are under a penalty of oxidative stress, which might adversely affect their health status.

## 8. Dietary Manipulation to Mitigate Heat-Stressed Rabbits

Several realistic strategies were used to alleviate the detrimental effects of rising temperatures, including dietary manipulation, which is becoming increasingly important in various parts of the world. Feed additives of nutraceuticals, including vitamins, minerals, antioxidants, probiotics, prebiotics, synbiotics, enzymes, organic acids, fatty acids, medicinal plants, etc., gain a great concern nowadays as available approaches to relieve the unfavorable impacts of HS via maintaining the common biological situation, strengthening the immune responsiveness, and preventing the illness that ultimately led to an increase in productivity.

### 8.1. Vitamins

#### 8.1.1. Vitamin C

Vitamin C (ascorbic acid) is a water-soluble antioxidant compound involved in protecting cells against oxidative stress and enhancing innate and adaptive immunity, as well as it is a vital co-factor in several enzymatic reactions. It is an effective antioxidant because it can donate electrons and enhance rabbits’ growth indicators under HS conditions and thermo-neutral temperatures [38,39,90]. Wang et al. [91] noted that dietary vitamin C (150–200 mg/kg diet) ameliorated the unfavorable influences of HS and enhanced the growth performance and liver/kidney function indicators in growing rabbits subjected to HS. Regarding its role as an effective antioxidant, vitamin C is involved in redox reactions, decreasing hypercholesterolemia development, suppressing free radicals’ production, and consequently, protracting cells from oxidative damage in rabbits under HS conditions [92]. Likewise, Hassan et al. [38] and Daader et al. [93] elucidated that dietary vitamin C (0.5 g/kg diet) enhanced growth performance and increased the serum concentration of SOD and TAC and reduced serum triglycerides, hydrogen peroxide, and MDA in heat-stressed growing rabbits. Hassan et al. [38] and Hassan et al. [90] explained that vitamin C enhanced the serum SOD activity via the upregulation of the mRNA of SOD.

Moreover, Abdel-Latif et al. [37] indicated that dietary vitamin C (200 mg/kg diet) improved the BWG and FCR and restored plasma concentrations of cortisol, leptin, IFN-γ, TNF-α, and IL-10 to normal levels compared to the HS group. They also proved that dietary vitamin C regulates the immune system via increasing the gene expression of HSP70 and forkhead box P_3_ (FOXP_3_) in liver and kidney tissues in NZW-fattening rabbits exposed to HS. From another point of view, Hassan et al. [38] documented that dietary supplementation of vitamin C decreased *Staphylococcus aureus*, *Escherichia coli*, and total bacterial counts in the cecum of growing rabbits. Furthermore, oral administration of ascorbic acid (2 mL of 300 ppm ascorbic acid), sodium bicarbonate (2 mL of 0.30% sodium bicarbonate), and its combination (2 mL mixture of 150 ppm ascorbic acid + 0.15% sodium bicarbonate) improved seminal antioxidative properties and minimized lipid peroxidation in heat-stressed rabbit bucks [94].

#### 8.1.2. Vitamin E

Vitamin E (α-tocopherol) plays a central role in several vital functions, such as cellular signaling, growth, reproduction, antioxidative status, immune response, and disease prevention [83,95]. Numerous studies reported that dietary vitamin E supplementation improved the growth performance of rabbits under HS conditions [40,93]. Sherif [40] demonstrated that dietary vitamin E (0.25 g/kg feed) enhanced the final BW, BWG, FCR, carcass yield %, and total edible parts % in rabbits subjected to HS conditions. Dalle Zotte et al. [14] observed that dietary vitamin E (200 mg/kg diet) increased the crude protein (CP), ether extract (EE), and total tract apparent digestibilities in broiler rabbits. These advancements may be attributed to the efficiency of vitamin E in protecting the integrity of small intestine mucosa and, consequently, enhancing the efficiency of digestion and absorption in the gut [96]. Interestingly, vitamin E is a potent intracellular antioxidant. Hashem et al. [97] noted that dietary vitamin E (150 mg/kg diet) increased plasma TAC and minimized LDL-cholesterol and MDA plasma concentrations. Additionally, dietary vitamin E improved the humoral immune response (antibody titers against SRBCs), enhanced plasma concentrations of GSH-Px and TAC, and minimized the plasma MDA concentration in growing rabbits [83]. Likewise, El-Ratel and Gabr [41] indicated that the dietary inclusion of vitamin E (100 mg/kg diet) improved pregnancy rates, litter size, serum antioxidative properties [GSH, GSH-Px, SOD, CAT, glutathione S-transferase (GST), and TAC] and immunity (lysozyme, IgG and IgM concentrations) in rabbits subjected to HS. Furthermore, adding vitamin E to drinking water decreased the serum cortisol concentration, ameliorating HS’ unfavorable influences on female rabbits’ productivity [98]. Regarding rabbit males, Sharaf et al. [99] pointed out that the administration of vitamin E in drinking water enhanced the semen quality characteristics (live sperm, normal sperm, volume, count, and motility) and decreased dead sperm, rectal temperature, skin temperature, ear temperature, and respiration rate under HS conditions, and these findings might be attributed to the antioxidative properties of vitamin E.

#### 8.1.3. Vitamin A

Vitamin A is vital in enhancing growth and maintaining a healthy eye, skin, mucus membranes, and immune system, including innate and acquired immunity [100]. It might be speculated that dietary vitamin A might relieve the unfavorable influence of HS in rabbits. It was postulated that vitamin A enhanced the activities of antioxidative enzymes, decreased lipid peroxidation, and improved the immune response in rabbits suffering from HS [101]. Recently, Yue et al. [42] postulated that adding vitamin A (12,000 IU/kg diet) ameliorated the negative impacts of HS on hair follicle development in Rex rabbits, as it increased the hair follicle density and fur quality. These improvements are connected with the role of vitamin A in regulating the Wnt10/β-catenin, IGF-I, fibroblast growth factor 5 (FGF5), noggin-BMP, and sonic hedgehog (SHH) signaling in rabbits under HS conditions. Vitamin A is available as feed supplements in retinyl acetate or retinyl palmitate (preformed vitamin A) and β-carotene (provitamin A). Vitamin A supplementation could be sufficiently met in rabbits by consuming β-carotene because intestinal mucosa can convert β-carotene to retinol [102]. Strychalski et al. [103] proved that the administration of Aztec marigold flower extract to rabbit feeds increased the gene expression of β-carotene oxygenase 2 (BCO_2_), which is involved in the metabolism of carotenoids and vitamin A. Moreover, dietary carotenoids increased the yellow pigmentation in rabbit meat, adipose tissue, liver, and milk. There is a high positive correlation between dietary carotenoids and their concentration in plasma, which is involved in enhancing the antioxidative properties [104,105]. Moreover, under experimental conditions, rabbits are often given large amounts of green food, covering the vitamin A requirements and enhancing their health status.

### 8.2. Minerals

#### 8.2.1. Selenium

Selenium (Se) is a crucial trace element needed for growth, reproduction, antioxidative status, and immune response via its involvement in the active site of GSH-Px. Studies postulated that different dietary sources of Se had a beneficial effect on growth performance and carcass characteristics in growing rabbits [6,43]. Ayyat et al. [7] revealed that the supplementation of rabbit diets with organic Se (0.03 mg/kg diet) enhanced the growth performance during mild temperatures and mitigated the adverse impacts of HS in the form of decreasing the rectal temperature, respiration rate, and heart rate during the summer season in growing rabbits. These results agree with Sheiha et al. [43], who demonstrated that the dietary supplementation of 25 and 50 mg of a nano-Se/kg diet enhanced the growth performance (live BW, BWG, FI, and FCR), carcass characteristics, liver/kidney functions, antioxidative status (GSH and SOD), and inflammatory cytokines (IFNγ and IL-4) in fattening rabbits suffering from HS. These results reflected the efficiency of Se dietary supplementation in enhancing rabbits raised in high ambient temperatures, enhancing productivity and health. Moreover, Se plays a specific role in the conservation of male fertility in the form of testosterone biosynthesis and the formation and development of spermatozoa. Several studies elucidated that dietary organic Se enhanced semen quality characteristics and reproductive performance of rabbit bucks under HS conditions [44,106]. Hosny et al. [44] documented that dietary 0.3 mg organic Se/kg diet decreased the rectal temperature, respiration rate, and seminal plasma concentration of MDA while increasing the total functional sperm counts, percentages of integrated sperm membranes, the seminal plasma concentration of TAC, kindling rates, litter size, and weight at birth in heat-stressed rabbits. On the other side, Abdulrashid and Juniper [107] postulated that a dietary organic Se supplementation had no significant impact on semen traits or the reproductive performance of buck rabbits under HS conditions. Sharaf et al. [98] revealed that a dietary combination of Se and vitamin E supported the synergism between them and increased the efficiency of alleviating the adverse impacts of HS on the productivity of female rabbits.

#### 8.2.2. Zinc

Zinc (Zn) is the second major prevalent trace element in the animal body. It is essential for regulating growth performance, immune response, antioxidative status, antibacterial activity, and gut health in mammals and birds [108,109]. Zn is connected with the structure and function of more than 300 enzymes responsible for synthesizing and degrading carbohydrates, proteins, lipids, nucleic acids, and antioxidant enzymes [110]. Zn could be provided as inorganic (ZnO), organic (Zn-methionine), and nano-Zn. Dietary Zn supplementation was proven to benefit the growth performance of fattening rabbits under HS conditions [16,111,112]. Under HS conditions, the dietary inclusion of ZnO (100 mg/kg; [112]) or nano-Zn (20, 40, 60, and 80 mg/kg; [16]) improved BW, BWG, FI, and FCR in growing rabbits. Similarly, Kamel et al. [111] observed that different dietary Zn sources (50 mg ZnO/kg diet or 30 mg nano-Zn/kg diet) enhanced the final BW, BWG, and FCR in growing rabbits who suffered from HS. Additionally, a dietary Zn addition increased the carcass characteristics, including hot carcass weight, dressing %, and total edible parts %, and reduced carcass fat % and meat lipid oxidation (meat TBARS content). At the same time, the water-holding capacity was improved [16]. El-Kholy et al. [45] showed that inorganic Zn (75 mg ZnSO_4_/kg diet) or organic Zn (75 mg Zn picolinate/kg diet) mitigated the adverse impacts of HS on the liver and kidney functions, antioxidative properties, and serum concentration of testosterone in NZW buck rabbits suffering from HS conditions. Dietary Zn supplementation positively impacted heat-stressed rabbits’ antioxidative properties and immune response [111,112]. Kamel et al. [111] elucidated that different dietary Zn sources (50 mg ZnO/kg diet or 30 mg nano-Zn/kg diet) elevated serum levels of GSH, GST, SOD, immunoglobulins (IgG and IgM), and high-density lipoproteins (HDL-cholesterol), while reducing serum concentrations of triglycerides and MDA in growing rabbits subjected to thermal stress conditions. These findings might be attributed to the role of Zn in activating the antioxidant enzymes and suppressing the ROS production and inflammatory responsiveness, which stimulate the immune response.

#### 8.2.3. Copper

Copper (Cu) is the most vital element of metalloenzymes involved in immune response, collagen synthesis, antioxidative status, bone formation, cardiac function, red blood cell formation, and rabbit metabolism [113]. Goodb et al. [46] noted that a dietary 200 mg Cu-methionine/kg diet or a 200 mg copper-glycine/kg diet enhanced the growth performance (BW, BWG, and FCR) and elevated serum concentrations of HDL-cholesterol, TAC, GSH-Px and SOD, while the total serum lipids and MDA were significantly reduced under hot summer conditions. Regarding its role in the antioxidative system, Cu is a vital component of SOD that is involved in scavenging harmful ROS. Additionally, it participates in several redox reactions, such as mitochondrial respiration (cytochrome oxidase) and energy metabolism [114]. Furthermore, Al-Sagheer et al. [47] showed that dietary Cu-acetate (100 mg/kg diet) and nano-Cu (50 mg/kg diet) improved the growth performance indicators (final BW, BWG, FI, and FCR), nutrients digestibility [nitrogen-free extract (NFE), DM and OM] and immunological parameters (serum contents of complement component-3, IgM, and lysozyme activity); however, carcass characteristics and carcass parts were not affected in growing rabbits. Cu greatly affects the immune system’s function, including enhancing Natural Killer cells’ and macrophages’ activities, regulating the proliferation and differentiation of T cells, and improving IL-2 production [115]. Regarding gene expression, Li et al. [116] observed that dietary Cu (39.1 mg/kg) supplementation is involved in up-regulating the gene expression of lipid metabolism, including carnitine palmitoyl transferases (CPT-1 and CPT-2) and peroxisome proliferator-activated receptor (PPAR-α), and down-regulating the gene expression of fatty acid synthase (FAS) and acetyl-CoA carboxylase (ACC) in the liver. Moreover, dietary Cu (39.1 mg/kg diet) supplementation up-regulated the gene expression of CPT-1, CPT-2, peroxisome proliferator-activated receptor-γ (PPAR-α), fatty acid transport protein (FATP), fatty acid-binding protein (FABP), and lipoprotein lipase (LPL) in skeletal muscles.

#### 8.2.4. Chromium

Chromium (Cr) is an essential element that plays a crucial role in glucose metabolism, lipids, proteins, and nucleic acids via promoting insulin activity, glucose transporter 4, and the glucose tolerance factor. Huang et al. [48] elucidated that adding organic Cr (0.4–1.6 mg/kg diet) enhanced the final BW, BWG, FI, and cell-mediated immunity in broiler rabbits suffering from HS. Cheng et al. [117] explained that dietary supplementation of diet Cr-yeast (0.8–1.0 mg/kg diet) ameliorated the adverse impacts of HS on spermatogenesis function and serum concentration of LH and FSH in rabbit bucks. Hashem and Al-Saadi [118] documented that the dietary supplementation of Cr-piclonat (300 and 500 pbb) enhanced the growth performance and reduced serum glucose and cortisone concentrations in rabbit males exposed to HS. Additionally, El-Kholy et al. [49] noted that the addition of Cr-yeast (2.5 mg/kg diet) improved the growth performance (final BW, BWG, FCR), serum testosterone level, and reproductive performance (sperm motility, alive sperm, and morphologically normal spermatozoa) in NZW rabbit males.

### 8.3. Electrolytes

HS increases respiration, disturbing the rabbits’ acid–base balance (respiratory alkalosis). Therefore, supplemental electrolytes, such as sodium bicarbonate (NaHCO_3_), potassium chloride (KCl), potassium bicarbonate (KHCO_3_), and ammonium chloride (NH_4_Cl), to the feed or drinking water might improve the acid–base balance in heat-stressed rabbits [39,94,119,120]. These buffers are involved in regulating the intracellular osmotic pressure and acid–base balance, and, consequently, promote heat tolerance. Anoh et al. [119,120] noted that administering an NaHCO_3_ and KHNO_3_ solution to drinking water maximized the ability to grow rabbits to resist HS, and they improved the FI and serum contents of calcium and T_4_. Ewuola et al. [94] demonstrated that the oral administration of NaHCO_3_ (2 mL of 0.30% NaHCO_3_) reduced respiratory and heart rates. At the same time, it improved seminal TAC and minimized lipid peroxidation in rabbit bucks subjected to HS. Abdel-Latif et al. [39] proved that treating heat-stressed rabbits with NaHCO_3_ (0.3 g/kg BW) improved the BWG and FCR, restored plasma concentrations of cortisol, leptin, IFN-γ, TNF-α, and IL-10 to normal levels, and decreased HSP70 mRNA and protein expressions in comparison to the HS group.

### 8.4. Probiotics

In recent years, probiotics (live beneficial microorganisms) received much attention as a safe alternative to antibiotics. Studies documented that probiotics administration improved rabbits’ growth rate, feed utilization, and gut health [18,50,75]. Moreover, probiotics positively impact nutrient digestibility and reduce the mortality rate in growing rabbits [121]. These enhancements could be achieved by: (1) establishing a healthy intestinal microbial equilibrium; (2) improving digestion and absorption of essential nutrients; (3) preserving the gut histomorphology, function, and health; (4) improving the antioxidative status; and (5) boosting the immune responsiveness [75,76]. Moreover, dietary probiotics inhibit the development and colonization of undesirable opportunistic pathogens and fortify the beneficial microorganisms that play a vital role in feed digestion, vitamin synthesis, volatile fatty acids (VFA) production, and antimicrobial peptide production [122]. Interestingly, dietary supplementation of probiotics ameliorated the unfavorable influences of HS in growing rabbits [50,51,75]. Fathi et al. [75] elucidated that rabbits fed 400 g/ton of *Bacillus subtilis* at 4 × 10^9^ CFU/g for 8 weeks enhanced their carcass characteristics (carcass weight, dressing %, and cuts of mid part and hind part as a percentage of live BW), improved meat chemical composition [dry matter (DM) %, organic matter (OM) %, CP %, and ash %], and enhanced the cell-mediated immunity in fattening rabbits under HS conditions. Likewise, Ayyat et al. [7] documented that the dietary addition of 1 g lactic acid bacteria (1 × 10^10^ CFU) or a 3 g yeast/kg feed improved the growth performance parameters, serum concentrations of hemoglobin, total protein, globulin, and albumin, while respiration rate, rectal temperature, and heart rate were decreased in growing rabbits reared in hot summer conditions. Hegab et al. [51] showed that the dietary supplementation of *Saccharomyces cerevisiae* (3 × 10^9^ CFU/kg) or *Lactobacillus acidophilus* (3 × 10^9^ CFU/kg) enhanced the growth performance indices supported by the proliferation of desirable yeast (*Yarrowia lipolytica*), and suppressed pathogenic bacteria (*Salmonella* spp., *Clostridium* spp., and *Enterobacteria* spp.) in NZW rabbits grown in HS conditions. Bassiony et al. [50] demonstrated that the addition of a 5 × 10^6^ CFU *C. butyricum*, 2 × 10^8^ CFU *Enterococcus faecium* NCIMB 11181, or 2.5 × 10^6^ CFU *C. butyricum* + 1 × 10^8^ CFU *E. faecium*/kg diet enhanced the growth performance (final BW, BWG, and FCR), immunological parameters (serum levels of total protein, globulin, HDL-cholesterol, complement component 3, and lysozyme activity), cecal fermentation (cecal concentrations of total VFA and butyric acids), and duodenal histomorphometry indices (higher villus height, thicker muscular layer thickness, lower crypt depth, and lower villus width) in broiler rabbits suffering from HS conditions. Additionally, Liu et al. [123] concluded that dietary *Clostridium butyricum*-enriched diets (1.0 × 10^5^ CFU/g) encouraged the growth performance by activating the digestive enzymes, enhancing intestinal morphology, supporting the gut flora colonization, and improving the immune response. The authors elucidated that the improvements in the immune response are connected with reducing the intestinal pro-inflammatory cytokines (IL-6, INF-γ, and TNF-α), the enhancement of gut barrier function via elevating secretory immunoglobulin A (sIgA) in the gut mucosa, and improving the relative expressions of MyD88, TLR2, and TLR4, as well as improving the intestinal antioxidative status throughout enhancing SOD, GSH-Px, and CAT and reduced MDA concentrations in the duodenum.

### 8.5. Prebiotics

Prebiotics (indigestible carbohydrates that encourage the growth and activity of beneficial intestinal flora) are a safe alternative to antibiotics as an immunostimulant. The prime commercial prebiotics are fructooligosaccharides (FOS), α-galactooligosaccharides (GOS), transgalactic-oligosaccharides (TOS), mannan-oligosaccharides (MOS), isomalto-oligosaccharide (IMO), and xilo-oligosaccharides (XOS) [124]. Ayyat et al. [7] pointed out that a dietary 3 g mannan-oligosaccharides/kg diet improved the growth performance parameters, serum levels of hemoglobin, total protein, globulin, and albumin, while the respiration rate, rectal temperature, and heart rate were decreased in growing rabbits reared under HS conditions. Abd El-Aziz et al. [19] revealed that dietary 0.3% MOS or 0.05% IMO improved the growth performance indicators (final BW and FI), carcass criteria (dressing % and giblets %), duodenal villi area and length, spleen white bulb area and length, liver GSH-Px, IGF-I, and SOD gene expression and muscle IGF-I receptor expression, while reducing the spleen IL-6 gene expression. Similarly, Abd El-Aziz et al. [20] observed that 0.5 or 1% FOS in drinking water enhanced growth parameters (final BW, BWG, FI, and FCR), carcass traits (total giblets %, liver %, and dressing %), serum antioxidant parameters (SOD, GSH-Px, and TAC), and cecal *Lactobacillus* count, while decreased the total bacterial count and *E. coli* populations in growing rabbits. Interestingly, Aboelhadid et al. [125] documented that drinking water supplemented with 2 g/L of Bio-Mos® minimized the predominance of *E. coli* and *Salmonella* connected with coccidiosis in fattening rabbits. Thus, Abdelhady and El-Abasy [126] concluded that the dietary prebiotic supplementation minimized the mortality rate and alleviated the negative clinical indications of the *Pasterella multocida* infection in rabbits.

### 8.6. Phytobiotics

The terms “phytobiotics”, “phytogenics”, or “medicinal plants” refer to a class of natural growth stimulants derived from plants, seeds, or herbs that contain biologically active substances and have a variety of biological effects. Thus, phytobiotics are being utilized more frequently in rabbit nutrition as antioxidants, physiological stimulants, flavorings, digestive aids, and colorants, and for the protection and treatment of various pathological disorders, as reviewed by Dalle Zotte et al. [127]. The focus on this point will be concentrated in the recent studies.

#### 8.6.1. *Moringa oleifera*

*Moringa oleifera* leaves (MOL) have attracted a lot of attention because of their great levels of nutrients and minimal anti-nutritional ingredients. The MOL might be able to prevent oxidation damage and display antioxidant properties that can combat the production of free radicals. Several studies documented that dietary MOL powder or extract could mitigate the unfavorable influences of HS in rabbits [23,52,53,81,128]. Khalid et al. [53] demonstrated that dietary MOL powder (200 mg/kg BW daily for 4 wk) enhanced te growth performance indicators, decreased rectal temperature and respiration rate, enhanced intestinal morphological parameters (increased jejunal weight, length, villus height, and villus height/crypt depth ratio), down-regulated the mRNA expression of TNF-α, HSP-A2, GSH-Px, and IL-1α, and elevated the expression of IL-6 in hyper-thermic rabbits. Additionally, Khalid et al. [53] elucidated that dietary MOL powder (200 mg/kg BW daily for 4 wk) enhanced the jejunal permeability (decreased jejunal contents of lipopolysaccharide, pro-inflammatory cytokines, and myeloperoxidase, while elevating the mRNA expression of tight junction proteins and GST activity, as well as reducing the MDA concentration in jejunal mucosa) and digestive function, positively modulate intestinal microbiota composition (increased beneficial genera including *Christensenellaceae* R-7 gut group, *Ruminococcaceae* NK4A214 group, *Ruminococcus* 2, *Lachnospiraceae* NK4A136 group, and *Lachnospiraceae*) and mucosal immunity (down-regulation of the gene expression of TNFRSF13C, LBP, and COX2, while up-regulating the gene expression of protein digestion and absorption pathways, such as PRSS2, LOC100349163, CPA1, CPB1, SLC9A3, SLC1A1, and SLC7A9) in heat-stressed rabbits. Similarly, Abdel-Latif et al. [39] reported that the dietary inclusion of 100, 200, and 300 mg/kg diet of MOL extract reduced the serum concentrations of cortisol, adrenaline, leptin, IFN-γ, TNF-α, alanine aminotransferase (ALT), aspartate aminotransferase (AST), urea, and creatinine, while increasing the serum IL-10 level as well as reducing the gene expression of HSP70 in the liver and kidney in NZW growing rabbits subjected to HS conditions. In heat-stressed NZW rabbits, Mutwedu et al. [23] noted that the administration of aqueous moringa seed extracts (200 mg/kg BW) enhanced the growth performance of pregnant and lactating females (FI, BW, and BWG), improved the reproductive performance (litter size from birth to weaning, litter weight, kid BW, and kid BWG), enhanced the reproductive hormones profile (serum concentrations of estradiol, progesterone, LH, and FSH), and improved ovarian histology. In heat-stressed V-line rabbit bucks, El-Desoky et al. [54] documented that oral supplementation of MOL ethanolic extract (50 mg/kg BW) improved heat tolerance (reduced rectal temperature), antioxidative status (increased serum TAC concentration), and semen quality (increased sperm concentration, sperm progressive motility, sperm viability, sperm normal morphology, intact acrosome sperm, and sperm with integrated cell membrane). Yasoob et al. [81,128] pointed out that dietary MOL powder (200 mg/kg BW daily for 28 d) elevated cecum weight %, enhancing cecal immunity (down-regulated the concentrations of cecal mucosa lipopolysaccharide and various inflammatory cytokines “TNF-α, IL-1α, IL-1β”), improved the expression of Nrf2, and up-regulating the antiapoptotic gene BCL2A1 (which may indicate protection from apoptosis), IL-6 family genes (which are responsible for immunity and survival), and PIK3R5 and TLR-2 (which are involved in thermo-tolerance), an improved cecal micro-ecosystem function (increased *Proteobacteria Actinobacteria*, and *Papillibacter* counts), reduced cecal lipid peroxidation (reduced cecal MDA), and down-regulating mucosal tissue inflammatory response (IL-10, IFN-γ, and RLA) in heat-stressed growing rabbits. 

#### 8.6.2. Ginger

Ginger (*Zingiber officinale*) is a medicinal plant with various health benefits as it contains several active phytochemical ingredients, including volatile oils, gingerol, gingerone, piperine, shogaols, zingerone, polyphenols, β-carotene, ascorbic acid, terpenoids, and alkaloids [129]. Dietary ginger relieved the unfavorable influences of HS on growing and adult rabbits. Amber et al. [55] proved that the addition of a 7.5 g ginger powder/kg diet enhanced the final BW, reduced the mortality rate, enhanced the FCR, increased the carcass weight, enhanced the digestibility of DM, OM, CP, and nitrogen-free extract (NFE), decreased the plasma levels of triglycerides, total cholesterol, HDL-cholesterol, and LDL-cholesterol, increased the plasma concentration of TAC, and reduced the plasma concentration of MDA in growing rabbits under HS conditions. Habeeb et al. [56] revealed that the dietary supplementation of ginger (250 mg/dose/d) reduced the rectal temperature, skin temperature, ear temperature, and respiration rate, improved BW and FI, enhanced the reproductive traits (conception rate, litter size, and litter weight at both birthing and weaning), increased plasma concentrations of progesterone, T_3_, T_4_, total protein, albumin, and globulin, and reduced the plasma levels of cortisol, urea, and creatinine of female rabbits under HS conditions. El-Ratel et al. [130] documented that the addition of ginger (200 mg/kg diet) improved the sexual desire (decreased reaction time and increased blood plasma testosterone), semen quality characteristics, fertility, and seminal plasma antioxidative status (increased TAC, GSH, and GST, while MDA was decreased) of rabbit bucks subjected to HS. Similarly, Ajao and Ola [131] showed that dietary ginger (15 g/kg feed) improved the semen quality characteristics (volume, motility, vitality, and abnormality) and serum TAC in rabbit bucks reared in hot, humid tropical conditions.

#### 8.6.3. Thyme

Thyme (*Thymus vulgaris*) is rich in active substances, including volatile essential oil phenolic acids and flavonoids, granting it several physical benefits, including antioxidant, immunomodulatory, antimicrobial, anti-inflammatory, antispasmodic, and anti-mutagenic properties [58,131]. Studies elucidated that the dietary addition of thyme, either as dried leaves (16 g/kg diet) or its essential oil (100 mg/kg feed), improved the appetite, growth performance, carcass parameters, and meat quality characteristics, while reducing the perirenal and scapular fat and meat lipid peroxidation in fattening rabbits under HS conditions [57,132]. Abdelnour et al. [58] stated that a dietary thyme essential oil (100 mg/kg diet) enhanced the antioxidative status (increased TAC and GSH, while decreased MDA), immunoglobulins (IgG and IgM), reproductive performance (ovulation rate, milk production, total litter size, live litter size at birth, litter size at weaning, viability rate at birth, and viability rate at weaning) in rabbit does subjected to HS. Abdel-Wareth et al. [133] elucidated that the dietary inclusion of thyme oil (500 mg/kg diet) enhanced the digestibility of nutrients (DM, CP, EE, and CF), liver functions (reduced serum concentrations of ALT and AST), semen quality traits (semen volume, sperm motility, vitality, and morphology as well as reduced abnormal sperm cells), and plasma testosterone concentration in rabbit males. Regarding the antioxidative and immunological properties of thyme, Yu et al. [134] indicated that thymol administration showed antioxidant activity and, consequently, a reduced oxidative stress and gene expression of IL-1β, IL-6, TNF-α, and TNF-β in hyperlipidemic rabbits. Similarly, adding thyme oil (0.5 g/kg diet) improved intestinal integrity, digestive efficiency, and antioxidative status while reducing the MDA concentration in rabbits’ duodenal mucosa [135]. 

#### 8.6.4. Turmeric

Turmeric (*Curcuma longa*) and its active compound, curcumin, have anti-inflammatory, anti-tumor, antioxidant, and other biological benefits [135,136]. El-Ratel et al. [59] reported that turmeric nanoparticles (2.5 g/kg diet) improved the growth performance (final BW, BWG, FCR, and viability), lipid profile (reduced plasma levels of total lipids, total cholesterol, triglycerides, LDL-cholesterol, while increased HDL-cholesterol), immunity (serum immunoglobulins), and antioxidant activity (TAC, SOD, GSH-Px, and GSH in plasma and hepatic tissues). In contrast, a reduced liver lipid peroxidation (MDA) in fattening rabbits suffered from HS. Similarly, Samy et al. [136] documented that dietary turmeric (curcumin) extract (200 or 400 ppm) enhanced growth performance (BW, BWG, and FCR), carcass characteristics (carcass weight and carcass %), meat chemical composition (increased protein, while decreased moisture and fat contents), nutrients digestibility (CP, CF, and EE), antioxidants status (increased TAC, GSH-Px, CAT, and SOD, while reduced MDA concentrations) in growing rabbits. In heat stressed-bucks, El-Kholy et al. [137] pointed out that dietary turmeric extract (30, 60, and 90 mg/kg diet) improved heat tolerance (decreased ear temperature and respiration rate), semen characteristics (increased mass motility, mitochondrial potential sperm %, normal sperm %, acrosome reacted sperm %, the total functional fraction of spermatozoa and epididymal weight %, while reduced germ cell apoptotic number, dead sperms %, and abnormal tail %), serum antioxidative properties (increased TAC and reduced MDA) and health status (reduced serum concentrations of total cholesterol and triglycerides) of rabbit males under HS conditions. Moreover, as a phytoestrogen, Sirotkin et al. [138] showed that dietary turmeric powder (5 g/100 kg feed) enhanced viability and fertility (number of liveborn and weaned pups), improved progesterone releasing, and stimulated ovarian follicles development during all folliculogenesis phases (increased primary follicles number and diameter of primary, secondary, and tertiary follicles) in adult rabbit does.

#### 8.6.5. Other Phytobiotics

Ragab et al. [24] reported that an oral dose of Maca (*Lepidium meyenii*) extract at 400 or 600 mg/rabbit twice weekly improved growth parameters (live BW, FI, and FCR), serum biochemical attributes (reduced serum concentration of total bilirubin, creatinine, glucose, total cholesterol, triglycerides, and AST, but enhanced cortisol and testosterone levels), antioxidant status (increase the serum concentration of TAC and SOD, while, MDA was reduced), and semen quality in rabbit bucks exposed to high ambient temperature. Additionally, in rabbits exposed to HS conditions, Ragab et al. [28] noted that an oral dose of Maca extract at 400 mg/kg BW/d improved the litter size, litter weights at 7, 14, 21, and 28 d, final BW, carcass characteristics (weights of dressing, thigh, trunk, liver, heart, and spleen), blood biochemical constituents (increased plasma levels of total protein, albumin, total bilirubin, and LDL-cholesterol, but decreased plasma contents of glucose, triglycerides, total cholesterol LDL-cholesterol, VLDL-cholesterol, uric acid, AST, and ALT), hormone profile (increased serum estrogen and progesterone concentration, while, cortisol concentration was reduced), antioxidative status (serum TAC concentration), and reproductive efficiency. Abdelnour et al. [60] elucidated that the dietary supplementation of pumpkin seed essential oil (2 mL/kg diet) improved the growth performance (live BW, FI, BWG, and FCR), serum biochemical parameters (increased serum levels of total protein, albumin, and globulin, while lowering plasma total bilirubin, ALT, AST, creatinine, uric acid, glucose, cortisol, T_3_, and corticosterone concentrations) and anti-inflammation agent (exhibiting negative caspase-3 immunoreactivity surrounding portal tract) in growing rabbits under HS conditions. Jimoh et al. [139] revealed that herbal supplements (10% Phyllanthus or 10% Mistletoe) enhanced semen quality traits (sperm concentration, motility, spermatozoa kinetics, and viability), reduced seminal lipid peroxidation, reduced serum corticosterone concentration and elevated serum concentrations of T_3_, LH, FSH, and testosterone in rabbits’ bucks reared under HS conditions. El-Bolkiny et al. [140] showed that daily oral dose of Sage leaves extracts (200 mg/kg BW), neem (50 mg/kg BW) or their combination reduced rectal temperature and improved BW, BWG, FCR, water intake, carcass weight, liver weight, heart weight, testes weight, and spleen weight in fattening rabbits suffered from HS conditions. El-Gindy et al. [141] elucidated that an oral dose of fresh onion juice (1.5–3 mL/kg BW) improved semen quality traits, elevated initial seminal fructose concentration, and antioxidant status in seminal plasma rabbit bucks exposed to severe HS. Jimoh et al. [84] noted that daily oral administration of soursop juice (2.22 mL/kg BW) enhanced the antioxidative status (serum concentrations of GSH, CAT, and SOD) and decreased lipid peroxidation (serum MDA concentration) in heat-stressed rabbit males.

## 9. Conclusions

HS is one of the main issues affecting rabbit productivity, particularly in hot and semi-hot regions of the world. In rabbits, HS has a negative impact on growth performance, meat quality, reproductive performance, antioxidative properties, immune responsiveness, intestinal histomorphology, and the microbiome. Nutraceuticals might be a viable procedure to relieve the unfavorable influences of HS and improve rabbit productivity during the hot season. Nutraceuticals are involved in improving the intestinal development, establishing a healthy intestinal microbial equilibrium, and enhancing the intestinal microstructure and immunity. It is interesting to note that nutraceuticals enhance the antioxidative characteristics and stimulate the immune system, which increases productivity and illness-resistance in harsh conditions. Additionally, nutraceuticals have a positive impact on meat’s chemical composition and meat physical quality characteristics. Getting such benefits are necessary for the rabbit industry, especially under stress conditions. More research is still needed to get a general recommendation about the dose of each nutraceutical under experimental conditions.

## Data Availability

Data is contained within the article.
